# Toward a Treatment of Cancer: Design and In Vitro/In Vivo Evaluation of Uncharged Pyrazoline Derivatives as a Series of Novel SHP2 Inhibitors

**DOI:** 10.3390/ijms23073497

**Published:** 2022-03-23

**Authors:** Jiajia Dai, Yiting Zhang, Yanan Gao, Xiaoyi Bai, Fang Liu, Shuo Li, Yanyan Yu, Wenpeng Hu, Ting Shi, Dayong Shi, Xiangqian Li

**Affiliations:** 1 State Key Laboratory of Microbial Technology, Institute of Microbial Technology, Shandong University, Qingdao 266200, China; daijiajia@sdu.edu.cn (J.D.); z17854265680@126.com (Y.Z.); dd37595252@163.com (Y.G.); 202112523@mail.sdu.edu.cn (X.B.); 202012681@mail.sdu.edu.cn (F.L.); 202012639@mail.sdu.edu.cn (S.L.); yuyanyan@sdu.edu.cn (Y.Y.); huwenpeng1202@163.com (W.H.); 2 College of Chemical and Biological Engineering, Shandong University of Science and Technology, Qingdao 266590, China; shiting_jia@126.com; 3 Laboratory for Marine Drugs and Bioproducts of Qingdao National Laboratory for Marine Science and Technology, Qingdao 266071, China

**Keywords:** anti-tumor, SHP2, inhibitor, apoptosis, toxicity

## Abstract

Src homology 2 domain-containing protein tyrosine phosphatase 2 (SHP2) is a non-receptor protein tyrosine phosphatase (PTP) encoded by the *PTPN11* gene, which is involved in the RAS/MAPK cell signaling transduction process. SHP2 has been shown to contribute to the progression of various cancers and is emerging as an important target for anti-tumor drug research. However, past efforts to develop SHP2 inhibitors into drugs have been unsuccessful owing to the positively charged nature of the active site pocket tending to bind negatively charged groups that are usually non-drug-like. Here, a series of uncharged pyrazoline derivatives were designed and developed as new SHP2 inhibitors using a structure-based strategy. Compound **4o**, which exhibited the strongest SHP2 inhibitory activity, bound directly to the catalytic domain of SHP2 in a competitive manner through multiple hydrogen bonds. Compound **4o** affected the RAS/MAPK signaling pathway by inhibiting SHP2, and subsequently induced apoptosis and growth inhibition of HCT116 cells in vitro and in vivo. Notably, the oral administration of compound **4o** in large doses showed no obvious toxicity. In summary, our findings provide a basis for the further development of compound **4o** as a safe, effective and anti-tumor SHP2 inhibitor.

## 1. Introduction

Src homology 2 (SH2) domain-containing protein tyrosine phosphatase 2 (SHP2) is a non-receptor protein tyrosine phosphatase (PTP) encoded by the *PTPN11* gene [1,2,3,4,5]. The SHP2 structure contains two tandem SH2 domains (N-SH2 and C-SH2), a PTP catalytic domain and a C-terminal tail with two tyrosine phosphorylation sites [6,7,8,9]. SHP2 is located in the cytoplasm, can be recruited by receptor tyrosine kinases (RTKs) to induce cell signaling and participates in the intracellular oncogenic RAS/MAPK cell signaling cascade [10,11,12,13,14]. SHP2 is involved in many diseases and is highly expressed in a variety of tumors including colon cancer, breast cancer, melanoma and lung adenocarcinoma [15,16,17,18,19,20]. Therefore, SHP2 is emerging as an important target for the treatment of cancer [21].

Given the importance of SHP2 as a potential anti-tumor target, the discovery of small molecule inhibitors of SHP2 has aroused widespread interest in the scientific community [22]. The development of small molecules targeting the PTP catalytic domain of SHP2 is a key approach for the development of SHP2 inhibitors [23]. Most of the catalytic site inhibitors of SHP2 contain ionic functional groups that can bind to the substrate catalytic pocket of SHP2 as a phosphotyrosine (pTyr) mimic to inhibit the activity of SHP2 [7,24,25].

Although there are several allosteric SHP2 inhibitors in clinical trials, such as JAB-3068, TNO155, RMC-4630 and RLY-1971, SHP2 catalytic site inhibitors have not been approved in clinical research at present [26,27]. The active binding site of pTyr is positively charged and tends to bind negatively charged groups such as sulfonic acid, carboxyl and nitro groups [7,25,28]. However, these non-drug-like groups often result in poor cell membrane permeability and oral bioavailability owing to the highly charged pharmacophores that are incorporated to achieve sufficient binding affinity [29,30]. Hence, few SHP2 catalytic site inhibitors have shown anti-tumor effects in vivo, which complicates drug discovery and development [31]. Based on this, novel uncharged small molecules might become the only avenue for the development of SHP2 inhibitors. 

Our group previously discovered that the catechol group in the marine derivative 3,4-dibromo-5-(2-bromo-3,4-dihydroxy-6-(isopropoxymethyl)benzyl)benzene-1,2-diol (HPN) could bind to the catalytic region of PTP1B [32]. SHP2 and PTP1B belong to the same family and have certain similarities in their catalytic regions [33]. Therefore, we attempted to target the catalytic region to design and synthesize a series of new uncharged catechol derivatives as SHP2 inhibitors to inhibit tumor growth in vitro and in vivo. Herein, the structure-based design and anti-tumor studies of potent uncharged SHP2 inhibitors will be presented.

## 2. Results

### 2.1. Design and Synthesis of SHP2 Inhibitors

**Figure 1:**
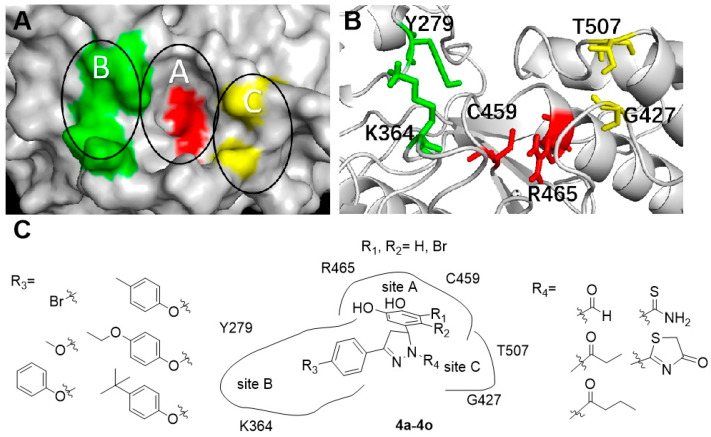
Design of SHP2 inhibitors. (**A**) Surface representation of the binding domain of SHP2 (front view). The main active region (site A), the second binding site (site B) and the third binding region (site C) were also labeled with A, B and C, respectively. (**B**) Positions of hotspot residues in a Ribbon diagram (side view). (**C**) Design strategy of catechol–pyrazoline derivatives as SHP2 inhibitors.

The main active region of SHP2 (site A) is the P-loop located at the bottom of the catalytic pocket, where Cys459 and Arg465 act as nucleophiles to recognize the phosphate group (Figure 1). The second binding site of SHP2 (site B) confers specificity to pTyr, which consists of some hydrophobic amino acid residues such as Lys364 and Tyr279. The third binding region (site C) is located at the other edge of the catalytic pocket, represented by Gly427 and T507.

Unlike most negatively charged groups, the electrically neutral catechol, which is the key group for HPN to occupy the catalytic region of PTP1B, was selected to occupy the catalytic region A of SHP2. This group could anchor the compounds by forming hydrogen bonds with amino acid residues such as Cys459, Lys366 and Arg465 at the bottom of the catalytic pocket. Para-substituted benzene ring groups (R_3_) were used as groups occupying site B to form p–π conjugation or π–π stacking while hydrophobic binding. Some small and flexible hydrophobic groups (R_4_) were chosen to occupy site C. A nonplanar 1,3,5-substituted pyrazoline ring was selected as the molecular skeleton of the target compounds **4a**–**4o**.

**Scheme 1:**
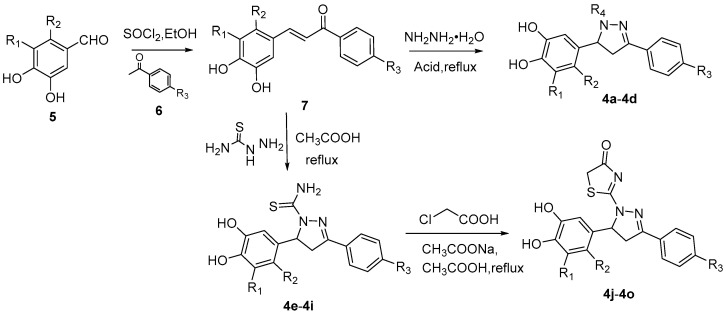
Synthesis of compounds **4a**–**4o**.

The synthetic routes of catechol–pyrazoline derivatives (**4a**–**4o**) are outlined in Scheme 1. Starting materials **5** and **6** were synthesized according to the reported methods [34]. Catechols **5** reacted with substituted acetophenones (**6**) under the action of SOCl_2_ in ethanol to generate intermediate product chalcones (**7**). Intermediate **7** and hydrazine hydrate were refluxed in different liquid acids (formic acid, propionic acid, butyric acid) to obtain the target products **4a**–**4d**, respectively. In addition, intermediates **7** were reacted with thiosemicarbazide to obtain carbothioamides **4e**–**4i** in acetic acid under reflux. Carbothioamides were cyclized with chloroacetic acid in acetic acid under reflux to afford target products **4j**–**4o**.

### 2.2. Inhibition of SHP2

SHP2 inhibition assay was conducted on synthetic ligands using the known method with sodium vanadate as a positive control [34,35]. As shown in Table 1, a series of compounds obtained by introducing groups such as thiazolone, thiocarboxamide, formaldehyde, 2-acetone and 2-butanone into the R_4_ moiety showed relatively different SHP2 inhibitory activities. The thiazolone group did confer better inhibitory activity to the compounds (**4l** vs. **4a** and **4e**, **4m** vs. **4b** and **4h**, **4k** vs. **4c**, **4d** and **4g**, **4j** vs. **4f**, **4n** vs. **4i**). When a group containing a benzene ring was introduced into the R_3_ moiety, the inhibitory activity of the compound against SHP2 was significantly improved. The para-substituents on the benzene ring have little effect on the activity. 

The inhibitory effect of compound **4o** (IC_50_ = 1.56 μM) on SHP2 was stronger than that of **4l**, which indicated that the bis-bromo-substituted catechol would increase the activity of the compound. In summary, when R_1_ and R_2_ are substituted with dibromine, R_3_ is introduced into a group with electron-donating conjugation effect, and R_4_ is introduced into thiazolone, the inhibitory activity of the compound will be obviously improved.

### 2.3. Compound 4o Is a Competitive SHP2 Inhibitor

**Figure 2:**
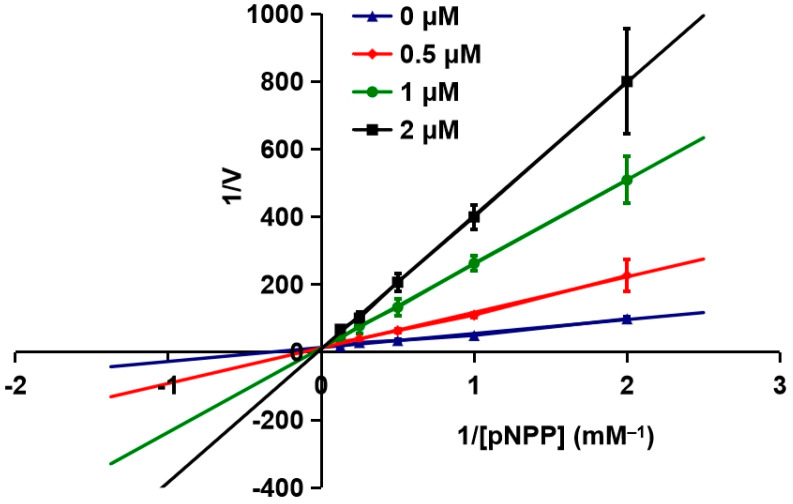
Inhibition kinetics study of compound **4o**. In the presence of various concentrations of **4o**, the reciprocal of initial reaction rate (1/[V]) was plotted against the reciprocal of pNPP concentrations (1/[pNPP]).

Inhibition kinetic studies were performed with various concentrations of pNPP in the absence or presence of inhibitors [25,32,34]. The experimental results were analyzed using double reciprocal plotting to evaluate the type of inhibition. As shown in Figure 2, the K_m_ value increased with the increasing concentrations of compound **4o**, whereas the V_max_ value was almost the same. The chart showed that straight lines intersected each other on the vertical axis, indicating that compound **4o** is a competitive SHP2 inhibitor.

### 2.4. Molecular Docking

**Figure 3:**
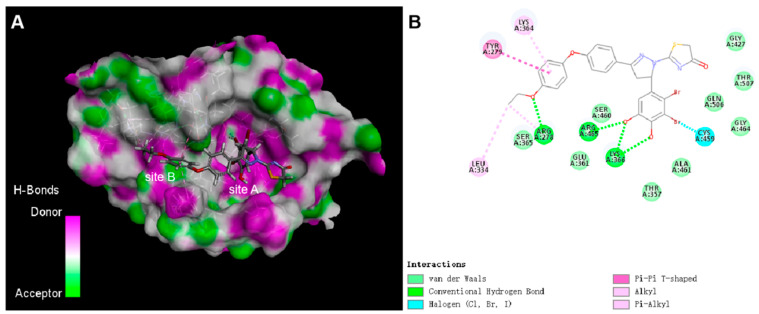
Molecular modeling of **4o**/SHP2 complex. (**A**) Predicted binding model of compound **4o** and SHP2 (PDB code 4RDD). (**B**) Schematic representation of the interactions between compound **4o** and SHP2.

The molecular docking study was performed using the BIOVIA Discovery Studio tool. The co-crystal structure of SHP2 with cefsulodin derivatives was chosen as the docking model (PDB code 4RDD). The surface binding mode between compound **4o** and SHP2 is shown in Figure 3A. The bromophenol moiety of the compound penetrated deep into the binding pocket of the catalytic site (site A), forming multiple critical hydrogen bonds with amino acid residues at the bottom of the pocket. The hydrophobic group at the para-terminal end of the benzene ring extended to site B through a slit, which stabilized the interaction between compound **4o** and SHP2.

As shown in Figure 3B, Lys366 and Arg465 around the catalytic site (Cys459) formed multiple traditional hydrogen bonds with the two hydroxyl groups of the bromophenol moiety of compound **4o**, which is the key active moiety for **4o** to exert SHP2 inhibition. These hydrogen bonds provide a stable anchoring site and significantly improve the binding stability between compound **4o** and SHP2. Meanwhile, the hydrophobic side chain part of the compound extended to the left shallow pocket to further stabilize the binding to the protein. The π–π stacking T conformation between the near-terminal benzene ring and Tyr279, the π–alkyl conjugation effect between the benzene ring and Lys364, the hydrogen bond between the oxygen atom of the terminal ethoxy group and Arg278, and the π–alkyl hyperconjugation effect formed by the terminal alkyl group with Arg278 and Leu334 significantly enhanced the binding stability of compound **4o** to SHP2.

### 2.5. Compound 4o Inhibits the Proliferation of Various Cancer Cells

**Figure 4:**
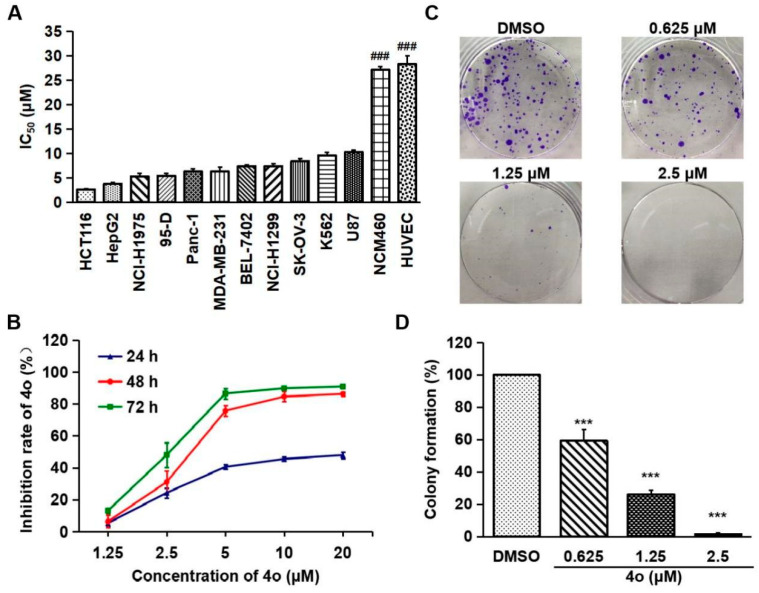
Compound **4o** inhibits various cells viability. (**A**) IC_50_ values of **4o** on cancer cells (HCT116, HepG2, NCI-H1975, 95-D, Panc-1, MDA-MB-231, BEL-7402, NCI-H1299, SK-OV-3, K562 and U87) and normal cells (NCM460 and HUVEC). Cells were treated with indicated concentration of **4o** for 72 h. Cell viability was determined by sulforhodamine B (SRB) or 3-(4,5-dimethyl-2-thiazolyl)-2,5-diphenyl-2-H-tetrazolium bromide (MTT) assay for K562 cells. (**B**) Cell viability inhibition rates (%) of **4o** on HCT116 cells. Cells were treated with indicated concentration of **4o** for 24, 48 and 72 h. Cell viability was determined by SRB assay. (**C**) Inhibition effect of **4o** on the colony formation of HCT116 cells. Cells were treated with **4o** (0.625, 1.25 and 2.5 μM) for 8 days and stained with crystal violet. Then, photos were taken and counted. (**D**) Quantification of the number of colonies. Colonies consisting of >50 cells were scored. Data are presented as means ± SD (*n* = 3). ^###^ *p* < 0.001 versus HCT116, *** *p* < 0.001 versus DMSO.

We first explored the viability inhibitory activity of compound **4o** against a variety of human cancer cells. As shown in Table 2 and Figure 4A, **4o** exhibited viability inhibitory activity against these cancer cells after 72 h of treatment. The IC_50_ values of **4o** were in the range of 2.64–10.25 μM. Notably, compound **4o** was significantly less toxic to normal cells, including NCM460 and HUVEC cells, than cancer cells, with IC_50_ values of 27.10 and 28.29 μM, respectively. Among these cancer cells, compound **4o** had the strongest inhibitory effect on the viability of HCT116 cells. Thus, HCT116 cells were selected for subsequent experimental research. A further assay found compound **4o** exhibited growth inhibition of HCT116 cells in a time- and dose-dependent manner (Figure 4B). The colony formation assay is the gold standard for detecting the effect of cytotoxic drugs on cancer cells in vitro [36]. Compound **4o** also significantly inhibited the colony formation of HCT116 cells (Figure 4C,D). These results demonstrate that compound **4o** has a broad anti-tumor activity and a favorable safety profile in vitro. 

### 2.6. Compound 4o Induces Apoptosis in HCT116 Cells

**Figure 5:**
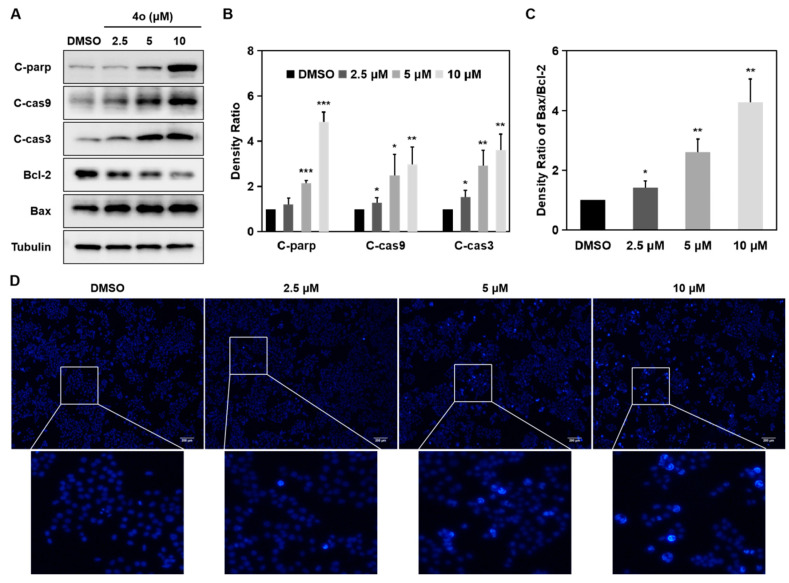
Compound **4o** induces HCT116 cells’ apoptosis. (**A**) Effects of **4o** on the expressions of proteins related to apoptosis. HCT116 cells were treated with **4o** (2.5, 5 and 10 μM) for 24 h. Protein levels were analyzed by Western blotting. (**B**,**C**) Protein band densities were quantified by normalizing to tubulin. (**D**) Changes in HCT116 cells’ nuclear morphology after **4o** treatment. HCT116 cells were treated with **4o** (2.5, 5 and 10 μM) for 24 h, stained with Hoechst 33,258 and visualized by fluorescence microscope (magnification, 40×). The bar represents 200 μm. Data are presented as means ± SD (*n* = 3). * *p* < 0.05, ** *p* < 0.01, *** *p* < 0.001 versus DMSO.

We further investigated whether the growth inhibition of HCT116 cells induced by compound **4o** was due to the induction of apoptosis. Caspase-9 is the apical caspase in the apoptotic pathway, and caspase-3 is the most important component of the caspase effector [37]. The cleavage and activation of caspase-9/caspase-3 are markers of cell apoptosis [38]. Furthermore, poly (adp-ribose) polymerase (parp) is a substrate for caspase-3, and its cleavage and activation are hallmarks of apoptosis [39]. As shown in Figure 5A–C, compared with DMSO, the levels of cleaved parp (C-parp), cleaved caspase9 (C-cas9) and cleaved caspase3 (C-cas3) in the HCT116 cells were increased after **4o** treatment for 24 h. Moreover, the ratio of the pro-apoptotic protein Bax to the anti-apoptotic protein Bcl-2 acts as a rheostat to determine the susceptibility of cells to apoptosis [40]. Compound **4o** caused a decrease in Bcl-2 and an increase in Bax. The expression of Bax/Bcl-2 ratio was up-regulated compared with DMSO.

In addition, we performed Hoechst 33,258 staining experiments. Chromatin pyknosis occurs with cells’ apoptosis, and their nuclei are denser and brighter [37]. As shown in Figure 5D, the nuclei were brighter after **4o** treatment than that of DMSO, suggesting that chromatin was condensed and apoptosis occurred. Taken together, these findings indicate that compound **4o** induces apoptosis in HCT116 cells.

### 2.7. Compound 4o Interacts with SHP2 and Suppresses RAS/MAPK Signaling Pathway in HCT116 Cells

**Figure 6:**
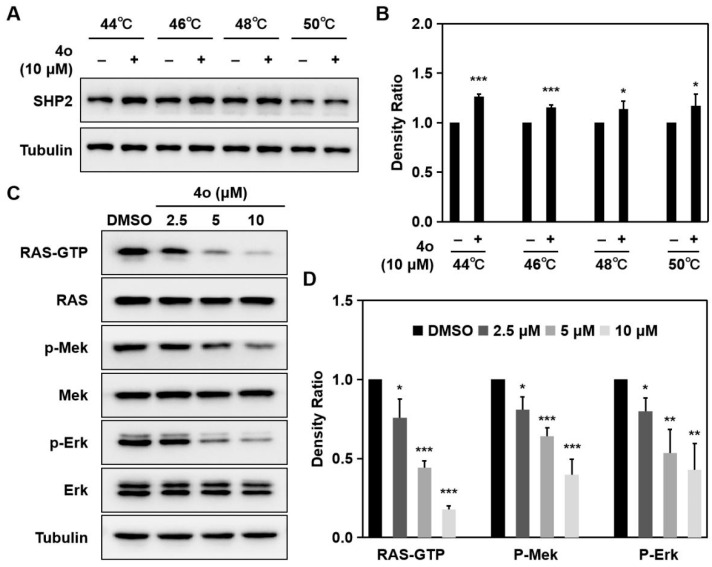
Compound **4o** interacts with SHP2 protein and suppresses RAS/MAPK signaling pathway in HCT116 cells. (**A**) Effect of **4o** on SHP2 protein stabilization. HCT116 cells were treated with 10 μM **4o** for 3 h before heated at different temperatures. Protein levels were analyzed by Western blotting. (**B**) Protein band densities were quantified by normalizing to tubulin. (**C**) Effects of **4o** on the expressions of Ras-GTP and p-Erk in HCT116 cells. Cells were treated with **4o** (2.5, 5 and 10 μM) for 12 h. Protein levels were analyzed by Western blotting. (**D**) Protein band densities were quantified by normalizing to RAS or Erk, respectively. Data are presented as means ± SD (*n* = 3). * *p* < 0.05, ** *p* < 0.01, *** *p* < 0.001 versus DMSO.

Cellular thermal shift assay (CETSA) can directly detect the mutual binding of the drug and target protein in cells [41]. When the drug molecule binds to the target protein in the cell, the thermal stability of the protein will be enhanced [42]. Therefore, we further evaluated whether compound **4o** could bind to SHP2 protein in HCT116 cells by the CETSA experiment. As shown in Figure 6A,B, compared to DMSO, the stability of SHP2 protein was obviously increased in HCT116 cells after **4o** treatment at the indicated temperatures, implying that compound **4o** directly interacts with SHP2 in HCT116 cells. 

SHP2 is involved in the regulation of the RAS/MAPK signaling pathway in cancer cells [43,44]. We further assessed the effect of compound **4o** on the RAS/MAPK signaling pathway in HCT116 cells. Compared with DMSO, the levels of RAS-GTP and p-Erk in HCT116 cells were decreased after **4o** treatment in a dose-dependent manner (Figure 6C,D). These results demonstrate that compound **4o** suppresses the RAS/MAPK signaling pathway by targeting SHP2 in HCT116 cells.

### 2.8. Compound 4o Induces Potent Anti-Tumor Activities In Vivo

**Figure 7:**
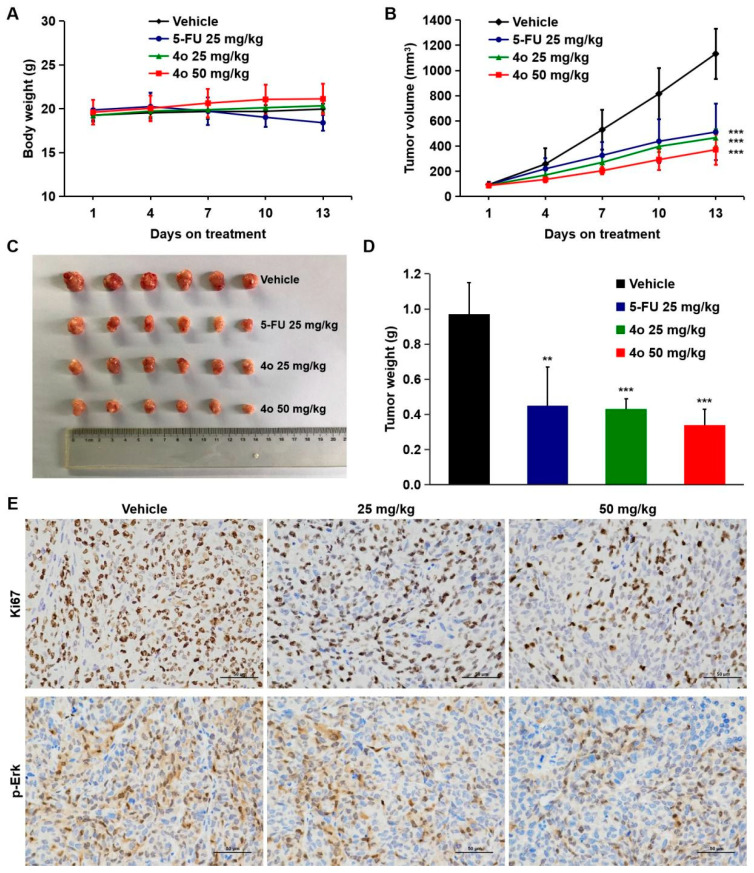
Compound **4o** induces potent anti-tumor activities in vivo. (**A**) Body weight of mice bearing subcutaneous HCT116 xenografts and administered either **4o** (25, 50 mg/kg), 5-FU (25 mg/kg) or vehicle (0.5% CMC-Na) for 13 consecutive days. (**B**) Anti-tumor efficacy of **4o** administered orally for 13 consecutive days at the doses and schedules indicated. (**C**) Representative photographs of the excised tumors from each group at 13 days of treatment. (**D**) Tumor weight of HCT116 after 13 days of treatment. (**E**) Ki67 and p-Erk expression levels in HCT116 tumors from each group were analyzed by immunohistochemical analysis. The pictures were captured at 400× magnification. The bar represents 50 μm. Data are presented as means ± SD (*n* = 6). ** *p* < 0.01, *** *p* < 0.001 versus vehicle.

As an SHP2 inhibitor, compound **4o** was confirmed to exhibit strong anti-tumor activity in vitro. Next, we explored the inhibitory effect of **4o** on tumor growth in vivo using a xenograft model of HCT116 cells. When the tumor volume reached approximately 100 mm^3^, compound **4o** was administered by gavage at doses of 25 and 50 mg/kg once daily, respectively. 5-fluorouracil (5-FU) is a chemotherapy drug commonly used in the treatment of colon cancer [45]. Therefore, 5-FU (25 mg/kg) was used as the positive drug. Compound **4o** did not have any obvious effect on the body weight of mice, while the 5-FU group showed a decrease, suggesting that compound **4o** is safe and of low toxicity (Figure 7A). As shown in Figure 7B, the xenograft tumors treated with **4o** developed obviously slower than those in the vehicle group. Tumors were isolated after the last drug treatment, and the results showed that compound **4o** significantly suppressed tumor size (Figure 7C), and tumor weight was much lighter (Figure 7D). We found that the tumor inhibition rates after 13 days of **4o** administrations were 58.8% and 67.2%, respectively, while that of 5-FU was 54.8% (Figure 7B).

The expression of Ki67 is closely related to the proliferation and growth of tumor cells, and is universally used in routine pathological examination as a proliferation marker [46]. We further assessed the levels of Ki67 and p-Erk using immunohistochemical assay. As expected, compound **4o** significantly decreased the expression of Ki67 and p-Erk (Figure 7E). These results indicate that compound **4o** inhibits tumor growth without obvious cytotoxicity in vivo.

### 2.9. Toxicological Safety Evaluation of Compound 4o In Vivo

**Figure 8:**
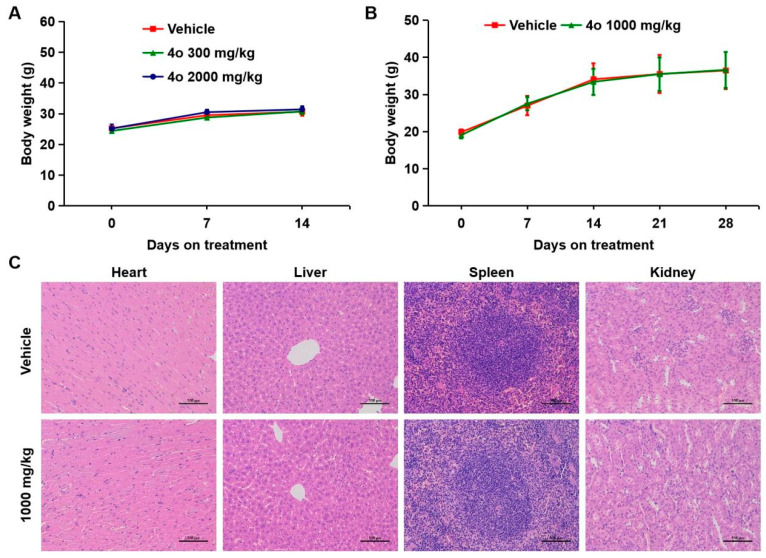
Toxicological safety evaluation of compound **4o** in vivo. (**A**) Body weight of mice treated with single **4o** (300, 2000 mg/kg) and vehicle (0.5% CMC-Na) for 14 days (*n* = 6). (**B**) Body weight of mice administered either **4o** (1000 mg/kg daily) or vehicle (0.5% CMC-Na) for 28 days (*n* = 10). (**C**) Histological analysis by H&E staining of heart, liver, spleen and kidney tissues in the mice treated with **4o** (1000 mg/kg, 28 days) and vehicle (0.5% CMC-Na). The pictures were captured at 200 × magnification. The bar represents 100 μm. Data are presented as means ± SD.

The above experiments have demonstrated that compound **4o** has excellent SHP2 inhibitory activity and clearly inhibits the growth of HCT116 cells in vitro and in vivo. We further performed acute and subacute toxicity experiments in mice to evaluate the safety of compound **4o** for oral administration. In acute toxicity experiments, we selected two drug concentrations, a low dose (300 mg/kg) and a high dose (2000 mg/kg). The mice were continuously observed and recorded for 14 days following a single oral administration. No obvious toxic effects, behavioral changes or mortality were found during the 14-day observation period in both dose groups. Compared with the vehicle group, there was no obvious decrease in the body weight of the mice in the administration group (Table 3, Figure 8A).

Subsequent subacute toxicity experiments in mice were carried out to observe the toxicity after continuous administration. The drug concentration was 1000 mg/kg, and the mice were orally administered for 28 consecutive days. Survival status and body weight changes in the mice were observed and recorded. As shown in Table 4 and Figure 8B, the body weights of the mice were not clearly suppressed after administration of **4o**. Additionally, no obvious toxic effects, behavioral changes or mortality were observed in the mice. H&E staining analysis of mice visceral tissue showed that there was no significant change in the cell morphology of heart, liver, spleen and kidney after 28 days of continuous administration (Figure 8C). The above results prove that compound **4o** has good safety in vivo.

## 3. Discussion

SHP2 is a prominent target for the discovery of anti-tumor drugs [47]. However, since the active site of SHP2 is positively charged and often binds to the non-drug-like negatively charged group, the development of SHP2 inhibitors into drugs in the past was not successful [31]. Therefore, novel uncharged pyrazoline derivatives as SHP2 inhibitors were designed and synthesized to overcome this difficulty. All of these derivatives exhibited SHP2 inhibitory activities in vitro, with IC_50_ values ranging from 1.56 μM to 34.17 μM. Kinetic analysis showed that compound **4o**, which exhibited the strongest inhibitory activity, was a competitive SHP2 inhibitor. The docking research proved the competitive inhibition pattern comprehensively, which was, compound **4o** bound to SHP2 competitively through the bromophenol group occupying the catalytic pocket and the hydrophobic long chain and thiazolone group occupying the peripheral region.

As expected, compound **4o** significantly inhibited the viability of various tumor cells, especially the human colon cancer cell line HCT116. The clone formation experiment further confirmed the inhibitory effect of compound **4o** on the proliferation of HCT116 cells. It is worth noting that the cytotoxicity of compound **4o** in normal cells was much lower than in tumor cells, implying that compound **4o** has certain safety in vitro. Apoptosis is the primary reason for growth inhibition [48]. Hoechst 33,258 staining analysis showed that compound **4o** induced chromatin condensation and apoptosis in HCT116 cells. Moreover, Western blot (WB) analysis indicated that compound **4o** up-regulated the levels of C-parp, C-cas3, C-cas9 and pro-apoptotic protein Bax and down-regulated the level of anti-apoptotic protein Bcl-2. These data demonstrate that compound **4o** may induce apoptosis of HCT116 cells via the mitochondrial apoptosis pathway. SHP2 mediates the RAS/MAPK signaling pathway to regulate cell growth and survival [49,50,51]. Compound **4o** was confirmed to be directly bound to SHP2 in HCT116 cells through a CETSA experiment. WB analysis further found that compound **4o** inhibited the activation of the RAS/MAPK signaling pathway by targeting SHP2. In other words, compound **4o** induced apoptosis and growth inhibition of HCT116 cells in vitro, which may be the result of RAS/MAPK pathway changes.

In view of the in vivo effects of numerous SHP2 inhibitors being inconsistent with their in vitro activities, we conducted in vivo studies to confirm the anti-tumor effects of compound **4o**. It is more important that oral availability is a challenge in the development of SHP2 inhibitors. Thus, we assayed the inhibitory effect of compound **4o** on tumor growth in vivo using a xenograft model of HCT116 cells. Long-term oral administration of compound **4o** significantly suppressed tumor growth in vivo. Notably, compound **4o** did not cause body weight decrease in mice, indicating that compound **4o** is safe and of low toxicity. 

The inherent electropositive property of the conserved phosphatase domain of SHP2 hinders the clinical development of SHP2 catalytic site inhibitors [8]. Sodium stibogluconate, the first compound reported to inhibit SHP2, was found to inhibit melanoma in vivo [52,53]. However, sodium stibogluconate showed severe adverse reactions during clinical research, including heart and liver toxicity, which limited its clinical application [54]. Different from sodium stibogluconate, compound **4o** showed no obvious toxicity in acute and subacute toxicity tests. Moreover, H&E staining analysis showed that compound **4o** had no significant toxicity to the heart, liver, spleen and kidney of mice, suggesting that compound **4o** has good safety in vivo. 

Tautomycetin was confirmed as an SHP2 catalytic site inhibitor (IC_50_ = 2.9 μM) [55]. Sulfophenyl acetic amide, an inhibitor targeting SHP2 (IC_50_ = 1.5 μM), showed an effective inhibitory effect on the growth of several cancer cells (H1975, MDA-MB-231 and SKBR3 cells) [25]. Compound 11g was also identified as an SHP2 catalytic site inhibitor (IC_50_ = 2.11 μM) [56]. Although these inhibitors showed inhibitory activity against SHP2 in vitro, no in vivo efficacy has been reported so far, which may be due to their inferior activity in vivo compared with in vitro. It is noteworthy that there are no reports of SHP2 catalytic site inhibitors entering clinical trials. This may be explained by the fact that the active site of SHP2, the pTyr binding domain, tends to bind negatively charged groups. However, the negative charge usually results in poor cell permeability and bioavailability, which seriously hinders the activity of these SHP2 inhibitors in vivo [57]. Unlike these inhibitors, compound **4o** is uncharged and showed potent anti-tumor activity and safety in vitro and in vivo. Therefore, compound **4o** exhibited high safety and in vivo activity, and is expected to break through the research bottleneck of SHP2 catalytic site inhibitors.

In conclusion, we utilized a structure-based approach to design a series of novel uncharged pyrazoline derivatives as SHP2 inhibitors. Compound **4o** suppressed the activation of the RAS/MAPK signaling pathway by inhibiting SHP2, and subsequently induced apoptosis and growth inhibition of HCT116 cells in vitro and in vivo without obvious toxic effects. These findings not only offer new chemotypes for anti-tumor drug development, but also provide a new perspective for the development of safe and potent SHP2 inhibitors.

## 4. Materials and Methods

### 4.1. Chemistry

All commercial reagents were purchased and used without further purification or distillation unless otherwise stated. NMR (^1^H, ^13^C) was obtained with Bruker AV-500 spectrometer with chemical shifts reported as parts per million (TMS as internal standard). The following abbreviations are used for multiplicity of NMR signals: s = singlet, d = doublet, t = triplet, q = quartet, m = multiplet. Column chromatography was performed on silica gel 200–300 mesh. Purity of all final products was determined by analytical HPLC to be >95%. HPLC purity of compounds was measured with a normal phase HPLC (Kromasil 100-5-C18, 10 × 250 mm) with two diverse wavelength detection systems. Compounds were eluted using a gradient elution of 40/60 to 20/80 H_2_O/CH_3_OH over 30 min at a flow rate of 2 mL/min.

### 4.2. Synthesis

General procedure for the preparation of 5-(3,4-dihydroxyphenyl)-3-(4-(4-ethoxyphenoxy)phenyl)-4,5-dihydro-1H-pyrazole-1-carbaldehyde (4a)

To 3,4-dihydroxybenzaldehyde (5a, 1.38 g, 0.01 mol) dissolved in ethanol (20 mL) was added 1-(4-(4-ethoxyphenoxy)phenyl)ethan-1-one (6a, 2.56 g, 0.01 mol). When complete solution was obtained, SOCl_2_ (0.5 mL) was incrementally added, while stirring. The reaction mixture was stirred at room temperature for 24 h. Then, 10 mL water was slowly added with rapid stirring under ice-water bath. The precipitate was filtered off and washed with ethanol. (*E*)-3-(3,4-dihydroxyphenyl)-1-(4-(4-ethoxyphenoxy)phenyl)prop-2-en-1-one (7a) was afforded as a yellow solid (2.6 g, 69.1%). To a mixed solution of 3 mL ethanol and 1 mL formic acid, 7a (188 mg, 0.5 mmol) and hydrazine hydrate (200 mg, 85%) were added with stirring. This mixture was heated at reflux for 2 h. After cooling to ambient temperature, the precipitate was filtered off and washed with water. Recrystallization of the crude product yielded the desired product 5-(3,4-dihydroxyphenyl)-3-(4-(4-ethoxyphenoxy)phenyl)-4,5-dihydro-1H-pyrazole-1-carbaldehyde (4a, 128 mg, 61.1%).

^1^H NMR (500 MHz, dmso) δ 8.92 (s, 1H), 8.84 (s, 1H), 8.82 (s, 1H), 7.74 (d, *J* = 8.7 Hz, 2H), 7.02 (d, *J* = 9.0 Hz, 2H), 6.95 (dd, *J* = 8.8, 5.3 Hz, 4H), 6.65 (d, *J* = 8.1 Hz, 1H), 6.56 (d, *J* = 1.8 Hz, 1H), 6.47 (dd, *J* = 8.1, 1.8 Hz, 1H), 5.31 (dd, *J* = 11.5, 4.1 Hz, 1H), 4.03–3.98 (m, 2H), 3.78 (dd, *J* = 18.0, 11.6 Hz, 1H), 3.10 (dd, *J* = 18.0, 4.2 Hz, 1H), 1.31 (t, *J* = 6.9 Hz, 3H). ^13^C NMR (126 MHz, dmso) δ 162.52 (s), 160.52 (s), 159.72 (s), 156.06 (s), 155.85 (s), 148.71 (s), 145.82 (s), 145.17 (s), 132.89 (s), 129.06 (s), 125.48 (s), 121.75 (s), 117.39 (s), 117.09 (s), 116.06 (d, *J* = 19.7 Hz), 113.22 (s), 63.83 (s), 59.83 (s), 42.84 (s), 15.13 (s). HRMS [M-H]: C_24_H_21_N_2_O_5_, calcd for 417.14560, found 417.14532.

Compounds 4b–4d were synthesized according to the synthetic method of compound 4a.

3-(4-(4-(tert-butyl)phenoxy)phenyl)-5-(3,4-dihydroxyphenyl)-4,5-dihydro-1H-pyrazole-1-carbaldehyde (4b)

^1^H NMR (500 MHz, dmso) δ 8.91 (s, 1H), 8.82 (overlap, 2H), 7.77 (d, *J* = 8.7 Hz, 2H), 7.43 (d, *J* = 8.7 Hz, 2H), 7.01 (t, *J* = 8.7 Hz, 4H), 6.65 (d, *J* = 8.1 Hz, 1H), 6.56 (d, *J* = 2.0 Hz, 1H), 6.48 (dd, *J* = 8.1, 2.0 Hz, 1H), 5.32 (dd, *J* = 11.5, 4.2 Hz, 1H), 3.80 (dd, *J* = 18.0, 11.6 Hz, 1H), 3.10 (dd, *J* = 18.0, 4.3 Hz, 1H), 1.28 (s, 9H). ^13^C NMR (126 MHz, dmso) δ 159.75 (s), 159.59 (s), 156.03 (s), 153.61 (s), 147.10 (s), 145.82 (s), 145.18 (s), 132.88 (s), 129.11 (s), 127.34 (s), 125.98 (s), 119.48 (s), 118.30 (s), 117.10 (s), 115.99 (s), 113.24 (s), 58.56 (s), 42.86 (s), 34.59 (s), 31.68 (s). HRMS [M-H]: C_26_H_25_N_2_O_4_, calcd for 429.18197, found 429.18173.

1-(5-(3,4-dihydroxyphenyl)-3-(4-phenoxyphenyl)-4,5-dihydro-1H-pyrazol-1-yl)propan-1-one (4c)

^1^H NMR (500 MHz, dmso) δ 8.86 (s, 1H), 8.76 (s, 1H), 7.77 (d, *J* = 8.4 Hz, 2H), 7.41 (t, *J* = 7.6 Hz, 2H), 7.18 (t, *J* = 7.4 Hz, 1H), 7.05 (t, *J* = 8.4 Hz, 4H), 6.63 (d, *J* = 8.0 Hz, 1H), 6.51 (s, 1H), 6.43 (d, *J* = 8.0 Hz, 1H), 5.33 (dd, *J* = 11.5, 3.7 Hz, 1H), 3.72 (dd, *J* = 17.9, 11.7 Hz, 1H), 3.03 (dd, *J* = 17.9, 3.8 Hz, 1H), 2.66 (q, *J* = 7.3 Hz, 2H), 1.04 (t, *J* = 7.5 Hz, 3H). ^13^C NMR (126 MHz, dmso) δ 170.78 (s), 158.93 (s), 156.28 (s), 153.94 (s), 145.71 (s), 144.90 (s), 134.08 (s), 130.65 (s), 128.99 (s), 126.79 (s), 124.56 (s), 119.65 (s), 118.77 (s), 116.81 (s), 115.95 (s), 113.06 (s), 59.55 (s), 42.44 (s), 27.38 (s), 9.50 (s). HRMS [M-H]: C_24_H_21_N_2_O_4_, calcd for 401.15796, found 401.15439.

1-(5-(3,4-dihydroxyphenyl)-3-(4-phenoxyphenyl)-4,5-dihydro-1H-pyrazol-1-yl)butan-1-one (4d)

^1^H NMR (500 MHz, dmso) δ 8.85 (s, 1H), 8.75 (s, 1H), 7.77 (d, *J* = 8.7 Hz, 2H), 7.41 (t, *J* = 7.9 Hz, 2H), 7.18 (t, *J* = 7.4 Hz, 1H), 7.05 (t, *J* = 7.7 Hz, 4H), 6.63 (d, *J* = 8.1 Hz, 1H), 6.51 (s, 1H), 6.43 (d, *J* = 8.0 Hz, 1H), 5.34 (dd, *J* = 11.6, 3.8 Hz, 1H), 3.72 (dd, *J* = 17.9, 11.7 Hz, 1H), 3.02 (dd, *J* = 17.9, 3.9 Hz, 1H), 2.64 (m, 2H), 1.57 (dd, *J* = 14.8, 7.4 Hz, 2H), 0.89 (t, *J* = 7.3 Hz, 3H). ^13^C NMR (126 MHz, dmso) δ 169.97 (s), 158.91 (s), 156.29 (s), 153.89 (s), 145.72 (s), 144.88 (s), 134.12 (s), 130.64 (s), 128.99 (s), 126.80 (s), 124.55 (s), 119.63 (s), 118.79 (s), 116.76 (s), 115.93 (s), 113.06 (s), 59.53 (s), 42.45 (s), 35.96 (s), 18.43 (s), 14.25 (s). HRMS [M-H]: C_25_H_23_N_2_O_4_, calcd for 415.17361, found 401.17496.

General procedure for the preparation of 5-(3,4-dihydroxyphenyl)-3-(4-(4-ethoxyphenoxy)phenyl)-4,5-dihydro-1H-pyrazole-1-carbothioamide (4e)

To an acetic acid (4 mL) solution of 7a (188 mg, 0.5 mmol) was added 54.6 mg (0.6 mmol) thiosemicarbazide with stirring. The mixture was heated at reflux for 4 h. After cooling to ambient temperature, 2 mL water was slowly added with rapid stirring under an ice-water bath. After standing for 1 h, the white solid that crystallized out of solution was filtered, washed with cold water and ethanol. Recrystallization of the crude product yielded the desired product 5-(3,4-dihydroxyphenyl)-3-(4-(4-ethoxyphenoxy)phenyl)-4,5-dihydro-1H-pyrazole-1-carbothioamide (4e, 140 mg, 31.2%).

^1^H NMR (500 MHz, dmso) δ 8.85 (s, 1H), 8.73 (s, 1H), 7.89 (s, 1H), 7.83 (d, *J* = 8.8 Hz, 2H), 7.74 (s, 1H), 7.06–6.98 (m, 2H), 6.94 (dd, *J* = 11.0, 9.0 Hz, 4H), 6.61 (d, *J* = 8.1 Hz, 1H), 6.47 (d, *J* = 2.0 Hz, 1H), 6.39 (dd, *J* = 8.1, 2.0 Hz, 1H), 5.70 (dd, *J* = 11.1, 2.8 Hz, 1H), 4.00 (q, *J* = 6.9 Hz, 2H), 3.76 (dd, *J* = 17.9, 11.2 Hz, 1H), 3.02 (dd, *J* = 17.8, 2.9 Hz, 1H), 1.31 (t, *J* = 7.0 Hz, 3H). ^13^C NMR (126 MHz, dmso) δ 176.11 (s), 160.51 (s), 155.79 (s), 155.04 (s), 148.78 (s), 145.55 (s), 144.70 (s), 134.49 (s), 129.47 (s), 125.69 (s), 121.64 (s), 117.36 (s), 116.80 (s), 116.12 (s), 115.86 (s), 112.97 (s), 63.83 (s), 62.89 (s), 43.01 (s), 15.13 (s). HRMS [M-H]: C_24_H_22_N_3_O_4_S, calcd for 448.13365, found 448.13315.

Compounds 4f–4i were synthesized according to the synthetic method of compound 4e.

5-(3,4-dihydroxyphenyl)-3-(4-methoxyphenyl)-4,5-dihydro-1H-pyrazole-1-carbothioamide (4f)

^1^H NMR (500 MHz, dmso) δ 8.83 (s, 1H), 8.70 (s, 1H), 7.84 (s, 1H), 7.80 (d, *J* = 8.5 Hz, 2H), 7.71 (s, 1H), 6.98 (d, *J* = 8.5 Hz, 2H), 6.62 (d, *J* = 8.0 Hz, 1H), 6.48 (s, 1H), 6.40 (d, *J* = 8.1 Hz, 1H), 5.72–5.68 (m, 1H), 3.79 (s, 3H), 3.78–3.70 (m, 1H), 3.04 (dd, *J* = 17.8, 2.4 Hz, 1H). ^13^C NMR (126 MHz, dmso) δ 176.00 (s), 172.44 (s), 161.58 (s), 155.39 (s), 145.55 (s), 144.68 (s), 134.56 (s), 129.25 (s), 123.93 (s), 116.84 (s), 115.86 (s), 114.59 (s), 113.02 (s), 62.80 (s), 55.82 (s), 43.03 (s). HRMS [M-H]: C_17_H_16_N_3_O_3_S, calcd for 342.09179, found 342.09122.

5-(3,4-dihydroxyphenyl)-3-(4-phenoxyphenyl)-4,5-dihydro-1H-pyrazole-1-carbothioamide (4g)

^1^H NMR (500 MHz, dmso) δ 8.84 (s, 1H), 8.72 (s, 1H), 7.90 (s, 1H), 7.87 (d, *J* = 8.4 Hz, 2H), 7.77 (s, 1H), 7.41 (t, *J* = 7.4 Hz, 2H), 7.18 (t, *J* = 7.3 Hz, 1H), 7.06 (d, *J* = 8.0 Hz, 2H), 7.02 (d, *J* = 8.5 Hz, 2H), 6.62 (d, *J* = 8.1 Hz, 1H), 6.49 (s, 1H), 6.41 (d, *J* = 8.0 Hz, 1H), 5.72 (d, *J* = 10.9 Hz, 1H), 3.78 (dd, *J* = 17.8, 11.3 Hz, 1H), 3.04 (d, *J* = 17.7 Hz, 1H). ^13^C NMR (126 MHz, dmso) δ 176.21 (s), 159.25 (s), 156.18 (s), 154.95 (s), 145.57 (s), 144.72 (s), 134.49 (s), 130.66 (s), 129.55 (s), 126.47 (s), 124.63 (s), 119.73 (s), 118.59 (s), 116.82 (s), 115.88 (s), 113.00 (s), 62.95 (s), 43.02 (s). HRMS [M-H]: C_22_H_18_N_3_O_3_S, calcd for 404.10744, found 404.10672.

3-(4-(4-(tert-butyl)phenoxy)phenyl)-5-(3,4-dihydroxyphenyl)-4,5-dihydro-1H-pyrazole-1-carbothioamide (4h)

^1^H NMR (500 MHz, dmso) δ 8.84 (s, 1H), 8.71 (s, 1H), 7.90 (s, 1H), 7.85 (d, *J* = 8.4 Hz, 2H), 7.75 (s, 1H), 7.42 (d, *J* = 8.4 Hz, 2H), 6.99 (t, *J* = 7.8 Hz, 4H), 6.62 (d, *J* = 8.1 Hz, 1H), 6.48 (s, 1H), 6.40 (d, *J* = 7.8 Hz, 1H), 5.71 (d, *J* = 11.1 Hz, 1H), 3.77 (dd, *J* = 17.8, 11.2 Hz, 1H), 3.03 (d, *J* = 17.8 Hz, 1H), 1.27 (s, 9H). ^13^C NMR (126 MHz, dmso) δ 176.18 (s), 159.54 (s), 154.98 (s), 153.72 (s), 147.00 (s), 145.57 (s), 144.71 (s), 134.49 (s), 129.51 (s), 127.31 (s), 126.22 (s), 119.31 (s), 118.33 (s), 116.80 (s), 115.87 (s), 112.99 (s), 62.92 (s), 43.02 (s), 34.58 (s), 31.68 (s). HRMS [M-H]: C_26_H_26_N_3_O_3_S, calcd for 460.17004, found 460.16956.

3-(4-bromophenyl)-5-(3,4-dihydroxyphenyl)-4,5-dihydro-1H-pyrazole-1-carbothioamide (4i)

^1^H NMR (500 MHz, dmso) δ 8.84 (s, 1H), 8.72 (s, 1H), 7.97 (s, 1H), 7.87 (s, 1H), 7.80 (d, *J* = 8.5 Hz, 2H), 7.63 (d, *J* = 8.5 Hz, 2H), 6.62 (d, *J* = 8.1 Hz, 1H), 6.48 (d, *J* = 1.9 Hz, 1H), 6.40 (dd, *J* = 8.1, 2.0 Hz, 1H), 5.73 (dd, *J* = 11.3, 3.0 Hz, 1H), 3.78 (dd, *J* = 18.0, 11.3 Hz, 1H), 3.06 (dd, *J* = 18.0, 3.1 Hz, 1H). ^13^C NMR (126 MHz, dmso) δ 176.53 (s), 154.41 (s), 145.58 (s), 144.75 (s), 134.40 (s), 132.12 (s), 130.78 (s), 129.42 (s), 124.35 (s), 116.83 (s), 115.89 (s), 113.00 (s), 63.15 (s), 42.77 (s). HRMS [M-H]: C_16_H_13_BrN_3_O_2_S, calcd for 389.99173, found 389.99127.

General procedure for the preparation of 2-(5-(3,4-dihydroxyphenyl)-3-(4-methoxyphenyl)-4,5-dihydro-1H-pyrazol-1-yl)thiazol-4(5H)-one (4j)

5-(3,4-dihydroxyphenyl)-3-(4-methoxyphenyl)-4,5-dihydro-1H-pyrazole-1-carbothioamide (172 mg, 0.5 mmol) was dissolved in acetic acid (10 mL). Chloroacetic acid (57 mg, 0.6 mmol) and sodium acetate (49.2 mg, 0.6 mmol) were successively added. The mixture was refluxed for 4 h. After cooling to room temperature, 1 mL water was slowly added with rapid stirring under an ice-water bath. After standing for 1 h, the white solid that crystallized out of solution was filtered, washed with cold water and ethanol. Recrystallization of the crude product yielded the desired product 2-(5-(3,4-dihydroxyphenyl)-3-(4-methoxyphenyl)-4,5-dihydro-1H-pyrazol-1-yl)thiazol-4(5H)-one (4j, 129 mg, 67.4%).

^1^H NMR (500 MHz, dmso) δ 8.98 (s, 1H), 8.89 (s, 1H), 7.77 (d, *J* = 8.8 Hz, 2H), 7.05 (d, *J* = 8.8 Hz, 2H), 6.66 (d, *J* = 8.1 Hz, 1H), 6.55 (d, *J* = 1.9 Hz, 1H), 6.48 (dd, *J* = 8.1, 2.0 Hz, 1H), 5.55 (dd, *J* = 11.0, 3.4 Hz, 1H), 3.97 (dd, *J* = 18.1, 11.1 Hz, 1H), 3.88 (s, 2H), 3.81 (s, 3H), 3.33–3.29 (overlap, 1H). ^13^C NMR (126 MHz, dmso) δ 187.22 (s), 177.05 (s), 162.30 (s), 160.79 (s), 145.87 (s), 145.54 (s), 131.93 (s), 129.56 (s), 122.74 (s), 117.28 (s), 116.09 (s), 114.92 (s), 113.15 (s), 63.81 (s), 55.93 (s), 43.91 (s), 39.13 (s). HRMS [M-H]: C_19_H_16_N_3_O_4_S, calcd for 382.08670, found 382.08636.

Compounds 4k–4o were synthesized according to the synthetic method of compound 4j.

2-(5-(3,4-dihydroxyphenyl)-3-(4-phenoxyphenyl)-4,5-dihydro-1H-pyrazol-1-yl)thiazol-4(5H)-one (4k)

^1^H NMR (500 MHz, dmso) δ 8.98 (s, 1H), 8.91 (s, 1H), 7.84 (d, *J* = 8.6 Hz, 2H), 7.43 (t, *J* = 7.7 Hz, 2H), 7.21 (t, *J* = 7.4 Hz, 1H), 7.09 (dd, *J* = 7.9, 6.4 Hz, 4H), 6.67 (d, *J* = 8.1 Hz, 1H), 6.55 (s, 1H), 6.49 (dd, *J* = 8.1, 1.6 Hz, 1H), 5.57 (dd, *J* = 11.0, 3.3 Hz, 1H), 4.00 (dd, *J* = 18.2, 11.1 Hz, 1H), 3.89 (s, 2H), 3.34–3.30 (overlap, 1H). ^13^C NMR (126 MHz, dmso) δ 187.26 (s), 177.33 (s), 160.48 (s), 160.12 (s), 155.88 (s), 145.88 (s), 145.57 (s), 131.86 (s), 130.73 (s), 129.88 (s), 125.12 (s), 124.89 (s), 119.98 (s), 118.66 (s), 117.28 (s), 116.10 (s), 113.16 (s), 63.93 (s), 43.92 (s), 39.16 (s). HRMS [M-H]: C_24_H_18_N_3_O_4_S, calcd for 444.10235, found 444.10181.

2-(5-(3,4-dihydroxyphenyl)-3-(4-(4-ethoxyphenoxy)phenyl)-4,5-dihydro-1H-pyrazol-1-yl)thiazol-4(5H)-one (4l)

^1^H NMR (500 MHz, dmso) δ 8.98 (s, 1H), 8.91 (s, 1H), 7.80 (d, *J* = 8.7 Hz, 2H), 7.04 (d, *J* = 8.9 Hz, 2H), 6.98 (dd, *J* = 12.2, 8.9 Hz, 4H), 6.66 (d, *J* = 8.1 Hz, 1H), 6.55 (d, *J* = 1.7 Hz, 1H), 6.48 (dd, *J* = 8.1, 1.8 Hz, 1H), 5.56 (dd, *J* = 11.0, 3.4 Hz, 1H), 4.03–3.95 (overlap, 3H), 3.88 (s, 2H), 3.32–3.27 (overlap, 1H), 1.31 (t, *J* = 6.9 Hz, 3H). ^13^C NMR (126 MHz, dmso) δ 187.24 (s), 177.25 (s), 161.30 (s), 160.53 (s), 155.94 (s), 148.54 (s), 145.88 (s), 145.56 (s), 131.88 (s), 129.80 (s), 124.41 (s), 121.78 (s), 117.51 (s), 117.27 (s), 116.18 (s), 116.10 (s), 113.15 (s), 63.88 (s), 63.86 (s), 43.92 (s), 39.15 (s), 15.13 (s). HRMS [M-H]: C_26_H_22_N_3_O_5_S, calcd for 488.12856, found 488.12784.

2-(3-(4-(4-(tert-butyl)phenoxy)phenyl)-5-(3,4-dihydroxyphenyl)-4,5-dihydro-1H-pyrazol-1-yl)thiazol-4(5H)-one (4m)

^1^H NMR (500 MHz, dmso) δ 8.98 (s, 1H), 8.90 (s, 1H), 7.82 (d, *J* = 8.8 Hz, 2H), 7.46–7.42 (m, 2H), 7.06 (d, *J* = 8.8 Hz, 2H), 7.03–7.00 (m, 2H), 6.66 (d, *J* = 8.1 Hz, 1H), 6.55 (d, *J* = 2.1 Hz, 1H), 6.48 (dd, *J* = 8.1, 2.1 Hz, 1H), 5.57 (dd, *J* = 11.0, 3.5 Hz, 1H), 4.02–3.96 (m, 1H), 3.89 (s, 2H), 3.33–3.28 (overlap, 1H), 1.28 (s, 9H). ^13^C NMR (126 MHz, dmso) δ 187.25 (s), 177.30 (s), 160.49 (s), 160.41 (s), 153.42 (s), 147.27 (s), 145.88 (s), 145.57 (s), 131.87 (s), 129.84 (s), 127.38 (s), 124.89 (s), 119.55 (s), 118.39 (s), 117.27 (s), 116.10 (s), 113.16 (s), 63.91 (s), 43.93 (s), 39.15 (s), 34.61 (s), 31.68 (s). HRMS [M-H]: C_28_H_26_N_3_O_4_S, calcd for 500.16495, found 500.16452.

2-(3-(4-bromophenyl)-5-(3,4-dihydroxyphenyl)-4,5-dihydro-1H-pyrazol-1-yl)thiazol-4(5H)-one (4n)

^1^H NMR (500 MHz, dmso) δ 8.98 (s, 1H), 8.92 (s, 1H), 7.76 (d, *J* = 8.6 Hz, 2H), 7.71 (d, *J* = 8.6 Hz, 2H), 6.67 (d, *J* = 8.1 Hz, 1H), 6.55 (d, *J* = 2.0 Hz, 1H), 6.49 (dd, *J* = 8.1, 2.1 Hz, 1H), 5.59 (dd, *J* = 11.2, 3.7 Hz, 1H), 4.01 (dd, *J* = 18.3, 11.2 Hz, 1H), 3.91 (s, 2H), 3.37–3.32 (overlap, 1H). ^13^C NMR (126 MHz, dmso) δ 187.34 (s), 177.76 (s), 160.17 (s), 145.88 (s), 145.60 (s), 132.50 (s), 131.74 (s), 129.59 (s), 129.53 (s), 125.48 (s), 117.33 (s), 116.11 (s), 113.23 (s), 64.13 (s), 43.73 (s), 39.21 (s). HRMS [M-H]: C_18_H_13_BrN_3_O_3_S, calcd for 429.98665, found 429.98630.

2-(5-(2,3-dibromo-4,5-dihydroxyphenyl)-3-(4-(4-ethoxyphenoxy)phenyl)-4,5-dihydro-1H-pyrazol-1-yl)thiazol-4(5H)-one (4o)

^1^H NMR (600 MHz, DMSO) δ 10.11 (s, 1H), 9.70 (s, 1H), 7.84–7.79 (m, 2H), 7.08–7.04 (m, 2H), 7.03–6.98 (m, 4H), 6.45 (s, 1H), 5.88 (d, *J* = 7.9 Hz, 1H), 4.25–4.10 (m, 1H), 4.04–4.01 (m, 2H), 4.01–3.95 (m, 2H), 3.26 (overlap, 1H), 1.34 (t, *J* = 7.0 Hz, 3H). ^13^C NMR (151 MHz, DMSO) δ 187.18 (s), 177.61 (s), 161.42 (s), 160.48 (s), 156.00 (s), 148.51 (s), 144.84 (s), 129.90 (s), 124.21 (s), 121.86 (s), 117.49 (s), 116.22 (s), 112.29 (s), 63.89 (s), 60.24 (s), 43.28 (s), 39.43 (s), 15.15 (s). HRMS [M-H]: C_26_H_20_Br_2_N_3_O_5_S, calcd for 645.94754, found 645.94720.

### 4.3. Protein Expression and Purification

pGEX-4T1 SHP2 WT (plasmid #8322) was purchased from Addgene. The SHP2 plasmid was transfected into E. coli BL21 (DE3) competent cells and cultured in LB medium containing 100 μg/mL ampicillin at 37 °C until the absorbance was 0.6 at 600 nm. Then, 0.2 mM IPTG (Sangon Biotech, Shanghai, China) was added to induce the expression of protein at 30 °C for 4 h followed by centrifugation and cells collection. Cells were then broken by ultrasound in lysis buffer (20 mM Tris–HCl (pH 8.0), 100 mM KCl, 10% glycerol, 1 mM DTT, 0.5% Triton X-100) on ice. The supernatant was collected by centrifugation at 10,000× *g* for 15 min at 4 °C. The BeaverBeads GSH (Beaver Biosciences Inc., Guangzhou, China) washed by lysis buffer were incubated with the supernatant for 2 h at 4 °C. Finally, the BeaverBeads GSH were eluted with elution buffer (100 mM Tris–HCl (pH 8.0), 100 mM KCl, 0.1% 2-mercaptoethanol, 20 mM GSH), and the supernatant was harvested to obtain the recombinant human SHP2 protein and stored at −80 °C.

### 4.4. SHP2 Inhibition Assay

The activity of SHP2 was determined using 4-nitrophenyl phosphate disodium salt (pNPP, Solarbio, Beijing, China) as the substrate. 4o (0.4, 1.2, 3.7, 11.1, 33.3, 100 µM) was preincubated with recombinant human SHP2 protein (240 nM) at 37 °C for 5 min. Then, the experiment was performed in a final volume of 100 μL containing 10 mM Tris–HCl (pH 7.5), 25 mM NaCl, 1 mM EDTA and 4 mM pNPP in a 96-well plate at 37 °C for 30 min. The amount of product p-nitrophenol was determined by the absorbance at 405 nm in a multimode reader (Berthold Technologies GmbH & Co.KG, Bad Wildbad, Baden Württemberg, Germany). The IC_50_ value was calculated by GraphPad Prism 7.0 software (GraphPad Inc, San Diego, CA, USA).

### 4.5. Inhibition Type of SHP2

The experimental process was similar to the SHP2 inhibition assay. In the assay, the initial rates at a series of pNPP concentrations were determined at various fixed inhibitor concentrations of 4o (0 µM, 0.5 µM, 1 µM and 2 µM) as described above. The values of 1/[S] and 1/[V] were obtained from the substrate concentration and initial reaction rate, and were plotted on the x-axis and y-axis, respectively. The inhibition type of SHP2 was determined according to the intersection characteristics of the obtained approximate line.

### 4.6. Molecular Docking

The crystal structure of the SHP2 (PDB code 4RDD) was obtained from the protein bank in the RCSB. The receptor protein was processed by PyMol, including removal of water molecules, addition of polar hydrogen atoms, and charge assignment. The 3D structures of ligands were generated using Chembio3D Ultra 11.0 followed by energy minimization. The CDOCKER module in Discovery Studio 2019 was used to perform molecularly docking. The receptor–ligand interactions were represented and analyzed on 3D and 2D diagram, respectively.

### 4.7. Cell Culture

Human colon cancer cell line HCT116, hepatoma cell lines HepG2 and BEL-7402, lung adenocarcinoma cell lines NCI-H1975 and 95-D, pancreatic cancer cell line Panc-1, breast cancer cell line MDA-MB-231, NSCLC cell line NCI-H1299, ovarian cancer cell line SK-OV-3, leukemia cell line K562, glioma cell line U87, colon epithelial cell line NCM460 and umbilical vein endothelial cell line HUVEC were purchased from the Institute of Biochemistry and Cell Biology, Chinese Academy of Sciences (Shanghai, China). HCT116 cell line was cultured in McCoy’s 5A medium (Hyclone, Logan, UT, USA). HepG2, 95-D, Panc-1, MDA-MB-231, BEL-7402, SK-OV-3, U87 and NCM460 cell lines were cultured in DMEM-High glucose medium (Hyclone, Logan, UT, USA). NCI-H1975, NCI-H1299 and HUVEC cell lines were cultured in RPMI 1640 medium (Hyclone, Logan, UT, USA). K562 cell line was cultured in IMDM medium (Hyclone, Logan, UT, USA). All of them were incubated in the medium with 10% fetal bovine serum (PAN, Adenbach, Bavaria, Germany) and 1% penicillin-streptomycin (Gibco-Invitrogen, Grand Island, NY, USA) under a humidified atmosphere of 5% CO_2_ at 37 °C.

### 4.8. Cell Viability Assay

The inhibition rate of cell viability was detected by 3-(4,5-dimethyl-2-thiazolyl)-2,5-diphenyl-2-H-tetrazolium bromide (MTT, Solarbio, Beijing, China) or Sulforhodamine B (SRB, Sigma-Aldrich, St. Louis, MO, USA) assay. In MTT assay, the suspension cells were inoculated in 96-well plates at the density of 5000 cells per well, and incubated with 4o at indicated concentrations dissolved in DMSO for 72 h. Each well was incubated with MTT solution (5 mg/mL) at 37 °C for 4 h, DMSO was added and incubated overnight at 37 °C. The absorbance at 570 nm was measured in a multimode reader (Berthold Technologies, Germany). In SRB assay, adherent cells were seeded in 96-well plates (5000 cells per well). After 24 h, the cells were treated with 4o for 72 h, and then fixed with precooling 10% TCA solution at 4 °C for 1 h. After washing and drying, SRB staining was added for 15 min, and then washed and dried by 1% acetic acid. Finally, Tris–HCl solution was added, and the absorbance at 515 nm was detected in a multimode reader (Berthold Technologies, Germany).

### 4.9. Colony Formation Assay

HCT116 cells were seeded in 6-well plates at a density of 500 cells per well. After 24 h, cells were treated with different concentrations of 4o for 8 days. Subsequently, the culture medium was removed and the cells were fixed with methanol for 3 min, and stained with crystal violet (Solarbio, Beijing, China) for 15 min. Finally, colonies were counted and photographed.

### 4.10. Western Blotting

HCT116 cells were harvested, lysed on ice in RIPA buffer (Beyotime Institute of Biotechnology, Shanghai, China) and boiled. Protein concentration was measured by BCA protein assay kit (Beyotime Institute of Biotechnology, Shanghai, China). The protein samples were separated by SDS-PAGE and transferred to the membrane (Pall, New York, NY, USA). Afterwards, the membrane was immunoblotted with the corresponding primary antibody (C-parp (#5625), C-cas9 (#9505), C-cas3 (#9661), Bcl-2 (#4223), Bax (#5023), SHP2 (#3752), P-Erk1/2 (#9101) and Erk1/2 (#9102), 1:1000, Cell Signaling Technology, Danvers, MA, USA; Tubulin (#ET1602-4), 1:5000, HUABIO, Hangzhou, China) and the appropriate HRP-conjugated secondary antibody, and determined with ECL detection reagent (Thermo Scientific, Waltham, MA, USA).

### 4.11. Hoechst 33258 Staining

HCT116 cells were treated with different concentrations of 4o for 24 h, then were stained with Hoechst 33,258 (Beyotime Institute of Biotechnology, Shanghai, China). The nuclear morphology was observed under fluorescence microscope.

### 4.12. CETSA

The assay was performed as described previously [58]. In brief, HCT116 cells were treated with DMSO or 4o for 3 h. Cells were resuspended in PBS and divided into four aliquots, and then the cell lysates were heated at different temperatures by T100^TM^ Thermal Cycler (Bio-Rad, Hercules, CA, USA). The heated cells were subjected to repeated freezing and thawing with liquid nitrogen for three times followed by centrifugation at 20,000× *g* for 20 min at 4 °C. Finally, the supernatants were gathered and boiled for 10 min after adding the loading buffer. Protein levels were assessed by Western blotting.

### 4.13. RAS Activation Assay

Active RAS (RAS-GTP) was pulled down from HCT116 cells. Cells were seeded in 6-well plates and treated with different concentrations of 4o for 12 h after overnight serum starvation. Cells were stimulated with 100 ng/mL hEGF for 5 min before cell lysis. On the basis of protein extraction and quantification, RAS activation assay biochem kit (Cytoskeleton Inc., Denver, CO, USA) was used for detection according to the requirements of manufacturers.

### 4.14. Anti-Tumor Activity Studies In Vivo

Male BALB/c-nude mice (Jinan Pengyue Experimental Animal Breeding Co., Ltd., Jinan, China), 5 weeks of age, were used. The mice were housed in a specific pathogen-free room with temperature controlled (22 ± 2 °C), humidity (55 ± 10%) and a light/dark cycle of 12 h, providing sterile food and water. The HCT116 cells xenograft model was conducted, and 1 × 10^7^ HCT116 cells were subcutaneously injected into the right armpit of mice. The tumor volume of mice was measured every three days to evaluate the tumor growth. When the tumor volume reached about 100 mm^3^, the mice were randomly divided into four groups (*n* = 6), and treated with vehicle (0.5% CMC-Na solution) or 4o (25, 50 mg/kg) by gavage once a day for 13 consecutive days. A total of 25 mg/kg 5-FU was used as a positive control. The body weight of mice was measured every three days. The length (L) and width (W) of tumors were measured by electronic caliper every three days. The volume (V) was calculated as follows: V = (L × W^2^)/2. When the tumor volume reached about 1000 mm^3^, the tumors were excised. Immunohistochemical analysis were used to evaluate the related protein levels.

### 4.15. Toxicological Safety Evaluation In Vivo

In acute toxicity assay, 18 female Kunming mice (Jinan Pengyue Experimental Animal Breeding Co., Ltd., Jinan, China), 18–22 g of weight, were used. The mice were randomly divided into three groups (*n* = 6), and treated with vehicle (0.5% CMC-Na solution) or 4o (300, 2000 mg/kg) by gavage once. The body weight, toxicity effect, general behavior and mortality of mice were monitored within 14 days after 4o treatment. In subacute toxicity assay, 10 male and 10 female Kunming mice (Jinan Pengyue Experimental Animal Breeding Co., Ltd., Jinan, China), 18–22 g of weight, were used. The mice were randomly divided into two groups with 5 males and 5 females, and were orally treated with vehicle (0.5% CMC-Na solution) or 4o (1000 mg/kg) once a day for 28 days. The body weight and mortality of mice were recorded within 28 days after administration of 4o. After 28 days of treatment, the mice were anaesthetized with isoflurane and killed by decapitation. The heart, liver, spleen and kidney tissues were collected, and histologically analyzed by H&E staining.

### 4.16. Statistical Analysis

Data were presented as mean ± SD. Statistical comparisons among groups were analyzed by Student’s *t*-test. These analyses were performed using GraphPad Prism 7.0 software (GraphPad Inc, San Diego, CA, USA). * *p* < 0.05, ** *p* < 0.01 and ** *p* < 0.001 were considered statistically significant.

## Figures and Tables

**Table 1:** Inhibition effect of pyrazoline derivatives (**4a–4o**) on SHP2 activities. Data are presented as means ± SD.

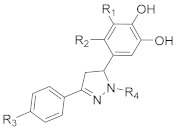					
Compound	R_1_	R_2_	R_3_	R_4_	IC_50_ (μM)
**4a**	H	H	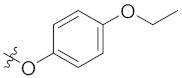	-CHO	13.33 ± 2.11
**4b**	H	H	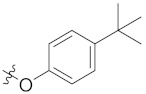	-CHO	13.74 ± 1.98
**4c**	H	H	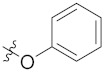	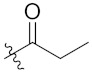	34.17 ± 5.90
**4d**	H	H	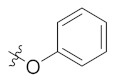	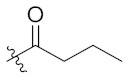	9.51 ± 2.01
**4e**	H	H	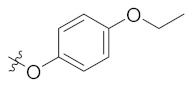	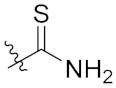	5.96 ± 0.92
**4f**	H	H	-OCH_3_	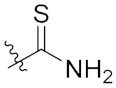	13.20 ± 1.89
**4g**	H	H	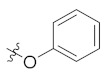	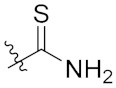	5.97 ± 0.88
**4h**	H	H	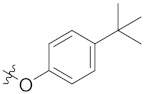	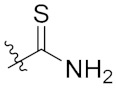	8.44 ± 2.03
**4i**	H	H	Br	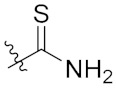	20.40 ± 2.45
**4j**	H	H	-OCH_3_	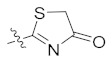	11.38 ± 1.56
**4k**	H	H	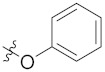	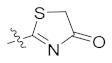	4.98 ± 0.77
**4l**	H	H	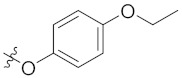	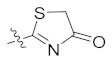	4.36 ± 0.65
**4m**	H	H	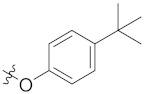	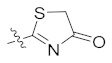	4.94 ± 0.79
**4n**	H	H	Br	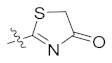	18.53 ± 2.32
**4o**	Br	Br	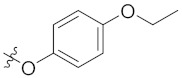	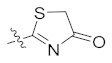	1.56 ± 0.25
**Na_3_VO_4_**					10.68 ± 2.08

**Table 2:** IC_50_ values of compound **4o** on various cancer cells and normal cells. Data are presented as means ± SD.

Cell Lines	IC_50_ (μM)
HCT116	2.64 ± 0.17
HepG2	3.80 ± 0.22
NCI-H1975	5.29 ± 0.60
95-D	5.42 ± 0.44
Panc-1	6.30 ± 0.50
MDA-MB-231	6.34 ± 0.37
BEL-7402	7.38 ± 0.31
NCI-H1299	7.42 ± 0.43
SK-OV-3	8.44 ± 0.45
K562	9.60 ± 0.68
U87	10.25 ± 0.39
NCM460	27.10 ± 0.66
HUVEC	28.29 ± 1.63

**Table 3:** Acute toxicity evaluation of compound **4o** in mice. Data are presented as means ± SD.

Groups	Mortality	Body Weight (g)		
		Day 0	Day 7	Day 14
Vehicle	0/6	25.25 ± 0.48	29.52 ± 1.06	30.67 ± 1.29
300 mg/kg	0/6	24.41 ± 0.64	28.78 ± 0.78	30.75 ± 0.99
2000 mg/kg	0/6	25.19 ± 1.45	30.51 ± 0.88	31.43 ± 1.09

**Table 4:** Subacute toxicity evaluation of compound **4o** in mice. Data are presented as means ± SD.

Groups	Mortality	Body Weight (g)				
		Day 0	Day 7	Day 14	Day 21	Day 28
Vehicle	0/10	19.87 ± 0.75	26.99 ± 2.56	34.08 ± 4.24	35.51 ± 5.15	36.44 ± 4.93
1000 mg/kg	0/10	19.08 ± 0.95	27.52 ± 1.77	33.41 ± 3.49	35.49 ± 4.52	36.63 ± 4.84

## Data Availability

Not applicable.
